# The Human Cutaneous Sensory Corpuscles: An Update

**DOI:** 10.3390/jcm10020227

**Published:** 2021-01-10

**Authors:** Ramón Cobo, Jorge García-Piqueras, Juan Cobo, José A. Vega

**Affiliations:** 1 Departamento de Morfología y Biología Celular, Grupo SINPOS, Universidad de Oviedo, 33003 Oviedo, Spain; ramoncobodiaz@gmail.com (R.C.); jgarciap@unizar.es (J.G.-P.); 2 Departamento de Anatomía e Histología, Universidad de Zaragoza, 50009 Zaragoza, Spain; 3 Instituto Asturiano de Odontología, 33006 Oviedo, Spain; jcobo@uniovi.es; 4 Departamento de Cirugía y Especialidades Médico-Quirúrgicas, Universidad de Oviedo, 33006 Oviedo, Spain; 5 Facultad de Ciencias de la Salud, Universidad Autónoma de Chile, Providencia 7500912, Santiago, Chile

**Keywords:** skin sensory innervation, sensory corpuscles, Meissner corpuscles, Ruffini corpuscles, Pacinian corpuscles, human

## Abstract

Sensory corpuscles of human skin are terminals of primary mechanoreceptive neurons associated with non-neuronal cells that function as low-threshold mechanoreceptors. Structurally, they consist of an extreme tip of a mechanosensory axon and nonmyelinating peripheral glial cells variably arranged according to the morphotype of the sensory corpuscle, all covered for connective cells of endoneurial and/or perineurial origin. Although the pathologies of sensitive corpuscles are scarce and almost never severe, adequate knowledge of the structure and immunohistochemical profile of these formations is essential for dermatologists and pathologists. In fact, since sensory corpuscles and nerves share a basic structure and protein composition, a cutaneous biopsy may be a complementary method for the analysis of nerve involvement in peripheral neuropathies, systemic diseases, and several pathologies of the central nervous system. Thus, a biopsy of cutaneous sensory corpuscles can provide information for the diagnosis, evolution, and effectiveness of treatments of some pathologies in which they are involved. Here, we updated and summarized the current knowledge about the immunohistochemistry of human sensory corpuscles with the aim to provide information to dermatologists and skin pathologists.

## 1. Introduction

**Figure 1:**
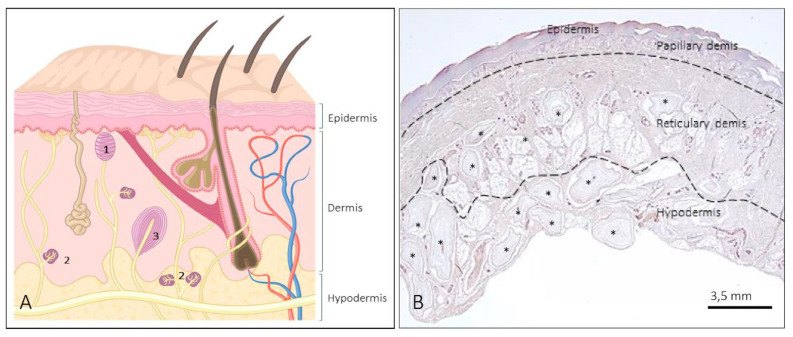
(**A**) Schematic representation showing the structure of the skin and the different morphotypes of sensory corpuscles. 1: Meissner corpuscles; 2: Ruffini corpuscles; 3: Pacini corpuscles; (**B**) low magnification of a histological section of human digital skin. The lines indicate the approximate limits of the three skin layers (epidermis, dermis—papillary and reticular—and hypodermis). Both the dermis and hypodermis contain abundant sensory corpuscles. * denotes Pacinian corpuscles.

**Figure 2:**
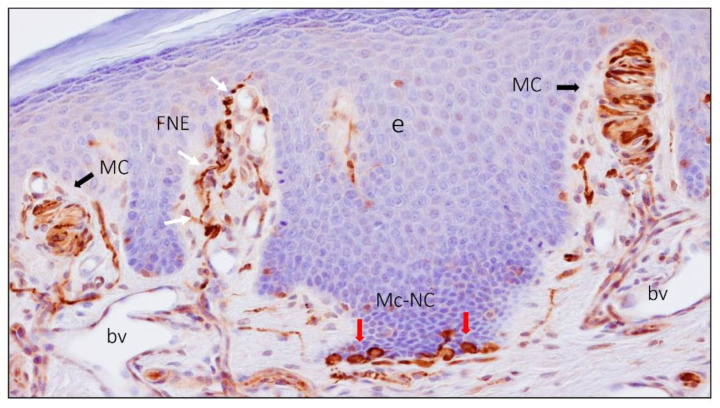
Section of human digital skin immunostained for the demonstration of neuron-specific enolase that labels axons in nerve fibers and sensory corpuscles as well as Merkel cells. FNE: free nerve ending (white arrows); MC: Meissner corpuscles (black arrows); Mc-NC: Merkel cell–neurite complex (red arrows). bv: blood vessels; e: epidermis.

The body surface of mammals is covered by two structurally different types of skin, i.e., nonhairy or glabrous and hairy skin. Glabrous skin contains no hairs, has a thick epidermal layer, and is restricted to zones characterized by high discriminative touch (shape, size, texture) such as the palms of hands or the plantar surfaces of the feet. Hairy skin covers more than 90% of the body surface, has a thin epidermal layer, and is strongly associated with affective touch [1]. Glabrous skin contains three types of sensory corpuscles, which have more or less specific tactile tuning properties: Meissner corpuscles, Ruffini corpuscles, and Pacinian corpuscles. Furthermore, it contains sensory Merkel cell–neurite complexes related to touch detection, but they are not sensory corpuscles sensu stricto (Figure 1 and Figure 2) [1,2,3,4]. These sensory structures work as low-threshold mechanoreceptors (LTMRs) in connection with a subpopulation of primary mechanosensory neurons whose bodies are localized in the dorsal root ganglia and the sensory ganglia of the cranial nerves [1,5]. LTMR nerve fibers are classified as Aβ, Aδ, or C based on their axon diameter, degree of myelination, and action potential conduction velocities [3,5]. Functionally, LTMRs fall into two categories, rapidly adapting (RA) and slowly adapting (SA) mechanoreceptors, which each have two variants: type I and type II [6,7]. Morphologically, they correspond with Meissner and Pacinian corpuscles (RAI and RAII, respectively), Merkel cell–neurite complexes (SAI), and dermal Ruffini corpuscles (SAII) [1].

The peripheral processes of Aβ LTMRs contact in the skin with specialized epithelial cells, i.e., Merkel cells, to form Merkel cell–neurite complexes (which are not included in this review) or with glial Schwann-like cells to form part of the sensory corpuscles [2,8]. Structurally, all cutaneous sensory corpuscles consist of a dendritic zone corresponding to the extreme tip of the peripheral process of an Aβ-LTMR surrounded by nonmyelinating glial cells variably arranged, both contained in a more or less developed capsule related to endoneurial/perineurial cells [9,10,11,12]. Thus, periaxonal cells are continuous with the cells of nerve trunks, demonstrating a relationship between nerves and the components that form sensory corpuscles [9,10]. Considering Pacinian corpuscles as a paradigm of the continuity between nerves and corpuscular components, the capsule and the outer core are continuous with the perineurium, the intermediate layer is related to the endoneurium, and the inner core corresponds to the Schwann cells [11,13]. Filling the spaces among cell layers is a chemically complex extracellular matrix, sometimes organized as a basal lamina [14,15,16,17,18].

In this review, we updated and summarized the current knowledge about the immunohistochemistry of human cutaneous sensory corpuscles with the aim to provide complete information to dermatologists and skin pathologists. The pathologies of cutaneous sensory corpuscles are rarely severe. However, detailed knowledge of their structure and immunohistochemical profile may be of interest because they can be quantitatively and/or qualitatively altered in some local pathologies (such as Dupuytren’s syndrome or neurofibromes) and systemic pathologies (diabetic neuropathy, human immunodeficiency virus neuropathy), and central nervous system diseases (among them, Parkinson’s disease, amyotrophic lateral sclerosis, and Guillain-Barré syndrome, as well as some psychiatric disorders and mental deficiencies) (for a review, see [19,20]). Consistently, cutaneous sensory corpuscles have gained interest since their biopsy can provide information on the diagnosis, evolution, and effectiveness of treatments of some of the pathologies in which they are involved [20,21,22,23]. Furthermore, because sensory corpuscles and nerves share a basic structure and protein composition, a cutaneous biopsy may be a complementary method for the analysis of nerve involvement in peripheral neuropathies, systemic diseases, and several pathologies of the central nervous system.

Although there are recent reviews on sensory corpuscles [1,10], none incorporate the new data related to their protein composition that occurred in the last 10 years, especially in humans. For this reason, we considered that it may be relevant to carry out an up-to-date review of the subject. In addition, this review is included in the Special Issue of the *Journal of Clinical Medicine* devoted to the “Structure and Function of Human Cutaneous Sensory Corpuscles” and is intended to be the starting point for a better understanding of other contributions.

The following sections detail the structure and protein composition of Meissner, Pacinian, and Ruffini corpuscles, as well as other less frequent morphotypes, of the human glabrous skin.

## 2. Meissner Corpuscles

Meissner corpuscles exist only in humans and primates, although Meissner-like corpuscles have been described in several mammal species [8]. They are typical of glabrous skin and are concentrated in skin areas especially sensitive to discriminative fine touch (fingertips, palm, soles of the feet, lips, and male and female genital skin) and occasionally on the tongue and palate [8]. They are located just below the epidermis, in the dermal papillae of the papillary dermis. Although their morphology and size are largely variable, they frequently have an oval shape with a larger axis perpendicular to the surface of the skin. Meissner corpuscles commence differentiation at around 20 weeks of estimated gestational age (wega), show basic morphology by 36 wega, and acquire a definitive aspect and immunohistochemical profile postnatally [24]. However, the density of Meissner corpuscles is not stable throughout their life, and their number and size are reduced with age [25].

**Figure 3:**
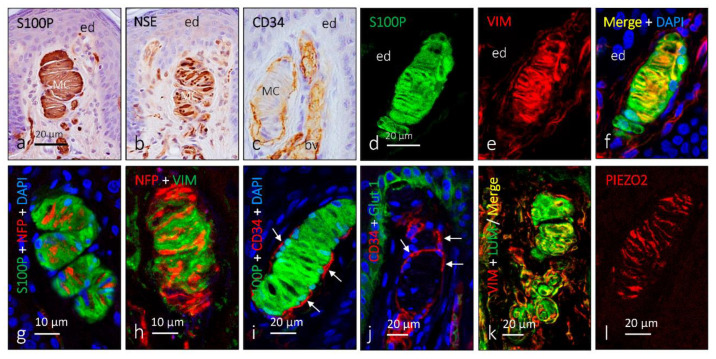
Immunohistochemical profile of human cutaneous Meissner corpuscles. Immunohistochemical localization of S100 protein (S100), neuron-specific enolase (NSE), CD34, Glut-1, vimentin (VIM), neurofilament protein (NFP), lumican (LUM) and PIEZO2 in human digital Meissner corpuscles. (**a**,**b**) Serial sections of the same corpuscle showing the lamellar cells (S100-protein-positive) and the axon (NSE-positive); (**c**) immunolabeling of the endoneurial capsule (CD34-positive); (**d**–**f**) images of double immunofluorescence demonstrating that S100P and VIM colocalize in lamellar cells. (**g**,**h**) variable relationships of the axon (NFP-positive) with the lamellar cells (S100P- or VIM-positive); (**i**,**j**) endoneurial, but not perineurial, nature of the capsule in Meissner corpuscles (CD34-positive; arrows); (**k**) expression of lumican, a component of the extracellular matrix, in association with the lamellar cells; (**l**) expression of PIEZO2, a mechanoprotein involved in mechanotransduction in the axon of Meissner corpuscles. ed: epidermis; MC: Meissner corpuscles. bv: blood vessels (in (**c**)).

**Figure 4:**
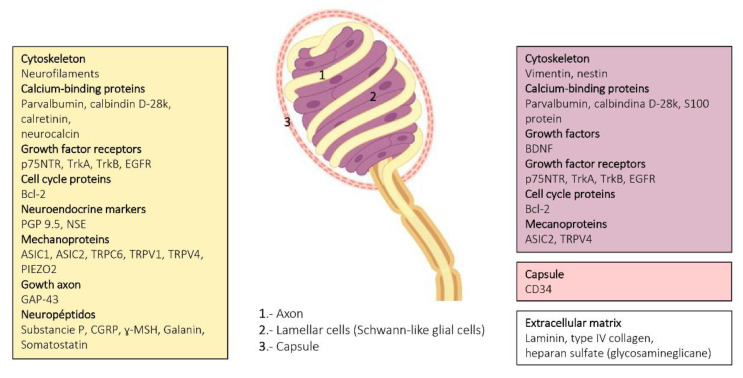
Schematic representation of Meissner corpuscles showing the proteins detected in the different corpuscular components using immunohistochemistry. Axon: yellow box, lamellar cells: purple box, capsule: pink box.

Meissner corpuscles consist of an axon, nonmyelinating glial cells (denominated laminar cells regarded as modified and specialized Schwann cells), and a capsule of fibroblasts of endoneurial origin (Figure 3 and Figure 4) [10,12]. The axon is usually unique, although, occasionally, one or two accessory axons can be found. In *Macaca fascicularis* and *Macaca mulata*, the main axon is an Aβ fiber, and the accessories are C or Aδ fibers [26]. Very recently, in mice, Neubarth and coworkers [27] observed that murine Meissner-like corpuscles are innervated by two mechanoreceptor subtypes that exhibit distinct responses to tactile stimuli. Whether or not this also occurs in humans remains to be demonstrated.

The myelin sheath that envelops the axon is lost upon entering the corpuscle [28], but the axon is always in relation to the laminar cells arranged as stacks of flattened sheets (classically described as a “coin stack”) usually arranged parallel to the skin surface. Between the laminar cells and the axon, there is an extracellular matrix of a very complex chemical composition [16,18]; isolating the corpuscle, there is a capsule of endoneurial nature [12].

Functionally, Meissner corpuscles are RA I-LTMRs that detect fine touch. For many years, they were exclusively related to the detection and discrimination of low-frequency vibration; however, they are also responsible for the detection of fine movements on the skin [29]. Furthermore, Meissner corpuscles have been proposed to function as nociceptors, as accessory axons express neuropeptides related to nociception [26], and evidence is being accumulated that not only axons but also lamellar cells are touch sensors [30].

## 3. Ruffini Corpuscles

**Figure 5:**
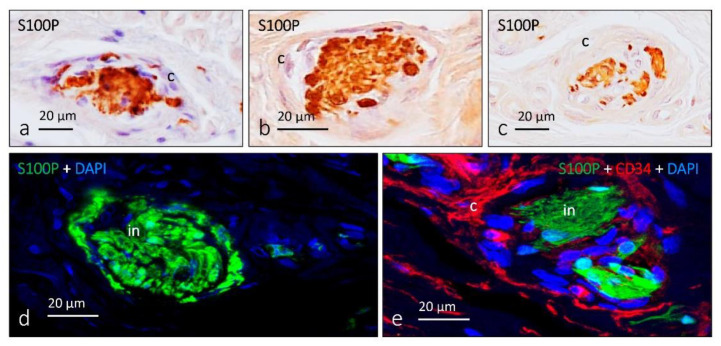
Immunohistochemical profile of human cutaneous Ruffini corpuscles. Immunohistochemical localization of S100 protein (**a**–**d**) in dermal Ruffini corpuscles shows different aspects depending on the section of the corpuscle, and the thickness of the capsule is also variable. (**e**) Surrounding the inner nucleus (S100-positive cells) of Ruffini corpuscles, there is a capsule (CD34-positive) of endoneurial nature; c: capsule; in: inner nucleus.

**Figure 6:**
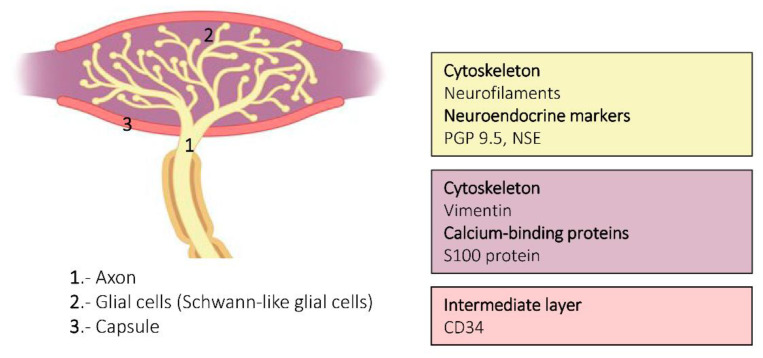
Schematic representation of Ruffini corpuscles showing the proteins detected in the different corpuscular components using immunohistochemistry. Axon: yellow box; lamellar cells: purple box; capsule: pink box.

Ruffini corpuscles (Figure 5 and Figure 6) or endings are elongated, spindle-shaped formations, with a length of up to 2 mm and a transverse dimension of 150 m in their central or equatorial portion and 40 m at extreme losses or poles. They are distributed in the dermis, ligaments, and joint capsules [1,2]. In human digital skin, they are scarce, with a density smaller than 0.3 corpuscles/mm^2^ [31].

Structurally, they consist of tree branches of a single axon embedded in peripheral glial cells without any organization and collagen fibers that have continuity in both poles of the extracorpuscular dermis. Surrounding and isolating the corpuscle is a capsule consisting of four or five layers of endoneurial cells [31,32].

Previously, Ruffini corpuscles were considered thermoreceptors, but recent evidence suggests that they could also play a role in detecting tactile stimuli (stretching, roughness) and represent II-LTMRs [1]. However, at least in humans, there is a mismatch between physiological and histological studies since, although electrophysiological studies demonstrate that glabrous skin shows abundant SA type II-LTMRs, histologically, they are very scarce. For example, in humans, a single Ruffini-like corpuscle was reported in the skin of the index finger, which is much less than what would be expected based on physiological recordings [7,33]. For this reason, the correlation between Ruffini corpuscles and slowly adapted mechanoreception has been questioned, and there are likely to be other nerve formations responsible or coresponsible for it [34].

## 4. Pacini (Pacinian) Corpuscles

**Figure 7:**
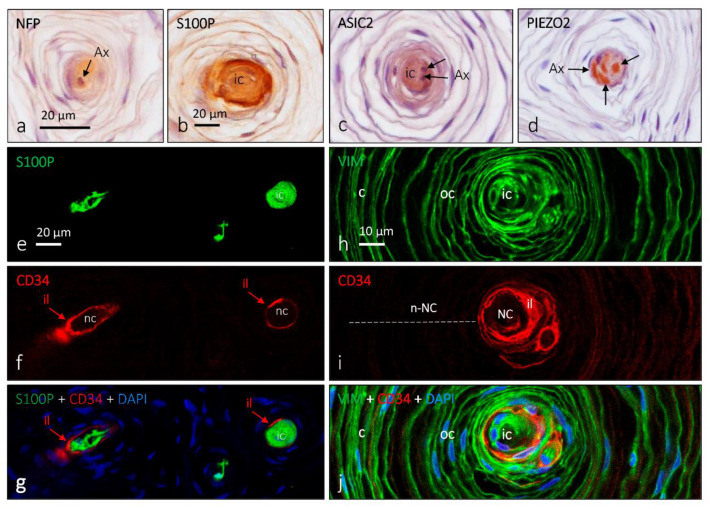
Immunohistochemical profile of human cutaneous Pacinian corpuscles. Immunohistochemical localization of neurofilament proteins (NFP), S100 protein (S100P), acid-sensing ion channel 2 (ASIC2), PIEZO2, CD34 and vimentin in human digital Pacinian corpuscles. Pacinian corpuscles are divided into two main compartments: the neural compartment (NC), which comprises the axon and the glial cells, and the non-neural compartment (n-NC), which comprises both the outer core and the capsule of perineurial origin. Both compartments are separated by an intermediate layer of endoneurial origin. All the Pacinian corpuscle constituents can be selectively immunolabeled. (**a**) The axon displaying immunoreactivity for NFP; (**b**) the inner core lamellae are S100P immunoreactive; (**c**,**d**) occurrence of the mechanoproteins ASIC2 and PIEZO2, respectively, in the axon; a faint immunoreactivity for ASIC2 is also present in the inner core; (**e**–**g**) double immunofluorescence, demonstrating that lamellar cells forming the inner core display the S100P and are covered by a layer of endoneurial-related cells (CD34-positive), the so-called intermediate layer; (**h**–**j**) double immunofluorescence, demonstrating that the lamellar system of Pacinian corpuscles (inner core, intermediate later, outer core, capsule) display vimentin and the division of Pacinian corpuscles into NC (within the CD34+ intermediate layer) and n-NC. Ax: axon; c: capsule; ic: inner core; il: intermediate layer; oc: outer core.

**Figure 8:**
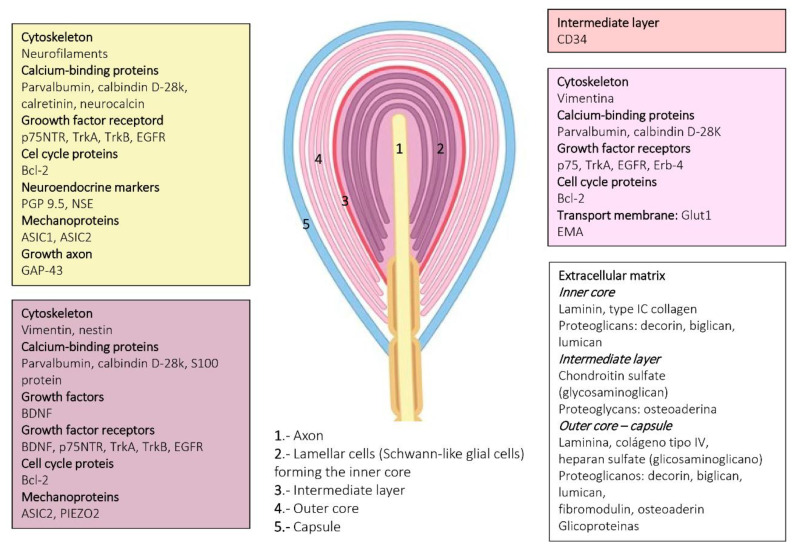
Schematic representation of Pacinian corpuscles showing the proteins detected in the different corpuscular components using immunohistochemistry. Axon: yellow box; lamellar cells: purple box; capsule: pink box; extracellular matrix: white box.

Pacini corpuscles are large, ovoid formations (up to 5 × 3 mm) largely distributed in most organs and tissues, including the deep dermis and hypodermis (Figure 1B, Figure 7 and Figure 8) [8,35]. In human digital skin, the development of Pacinian corpuscles begins at 13 wega and is completed at four months of life, although their basic structure and immunohistochemical characteristics are reached at 36 wega. During development, around the axon, a complex network of S100-positive Schwann-cell-related processes is progressively compacted to form the inner core, and the surrounding mesenchyme organizes to form the outer core and the capsule [24]. On the other hand, Pacinian corpuscles generally show no relevant age-related alterations [25].

Cells forming Pacinian corpuscles are typically arranged around the axon, showing a typical appearance of an “onion bulb” (Figure 7 and Figure 8). Within the laminar formations can be distinguished two compartments called the inner core and the outer core; both are surrounded by a fibrous capsule of varying thickness. In the central part of the inner core is the axon of an Aβ-LTMR, which is directly surrounded and attached to nonmyelinating Schwann-like cells denominated laminar cells (neural compartment) [35]. Among these lamellae is a molecularly complex extracellular matrix [17,18]. Occasionally, Pacinian corpuscles contain additional C or Aδ fibers, which are thought to be sensory or postganglionic sympathetic (see [36]).

The non-neural compartment of the corpuscles is divided into an outer core and a capsule. The outer core consists of flattened fibroblast-like cells, which completely surround the inner core. Between the inner and the outer cores, there is an intermediate cell stratum, the cellular elements of which are modified endoneural fibroblasts [11]. Finally, on the outside of the corpuscle, there is a capsule of variable thickness, which, like the outer core, comes from the perineurium. Typically, the capsule contains capillaries and macrophages.

Functionally, Pacini corpuscles are representative of RA II-LTMRs and respond to pressure and vibratory stimuli between 20 and 1500 Hz, with a maximum sensitivity at 200–400 Hz [6,7,34].

## 5. Other Morphotypes of Sensory Corpuscles

**Figure 9:**
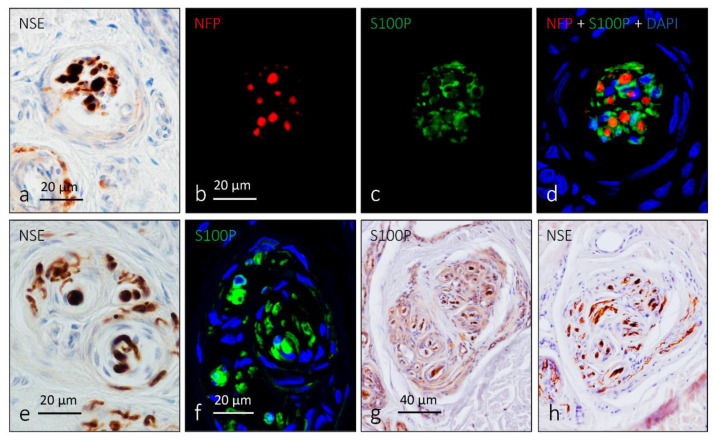
Immunohistochemical localization of neurons specific enolase (NSE), neurofilament protein (NFP) and S100 protein (S100P) in possible Krause corpuscles (**a**–**d**), Golgi–Mazzoni corpuscles (**e**,**f**) and a cluster of small lamellar corpuscles (**g**,**h**). Axons display strong immunoreactivity for NSE and NFP, and Schwann-like cells are S100P-positive. (**b**–**d**) double immunofluorescence demonstrating NFP- and S100P-immunoreactivity in axons and glial cells, respectively in possible Krause corpuscles.

In addition to the above-mentioned main types of cutaneous sensory corpuscles, other morphotypes have been identified in the skin. Corpuscles receptors defined by light microscopy include Krause end-bulbs and Golgi–Mazzoni corpuscles (Figure 9).

Krause corpuscles or bulbs are encapsulated sensory corpuscles occurring in the skin and mucous membranes, previously regarded as sensory cold receptors. Structurally, they present in glabrous skin and are made up of axon endings sheathed by Schwann-like cells contained in a fine fibroblastic capsule. Occasionally, they have additional thin axons with dense-core vesicles. Many authors consider Krause end-bulbs as clusters of free nerve endings.

The term Golgi–Mazzoni corpuscles has been used to describe a similar structure to Pacinian corpuscles found only in the fingertips. They consist of completely concentric lamellae resembling small Pacinian corpuscles and have also been called simple corpuscles. They have few lamellae in the outer core–capsule system, and the asymmetrical position of the inner core cells around the axon tip is a typical characteristic of this kind of mechanoreceptor. They differ from Pacinian corpuscles because of their smaller dimensions, surface position in the dermis, considerably thinner capsule, the frequent presence of two afferent myelinated nerve fibers, and the absence of capillaries on the capsule [1,2,3].

## 6. Proteins in Human Sensory Corpuscles

Cutaneous sensory corpuscles have a very complex and heterogeneous protein content, as demonstrated in numerous studies performed for more than 40 years based on immunohistochemical data. In recent years, the study of Meissner corpuscles and, to a lesser extent, other kinds of cutaneous sensory corpuscles, was added to the study of peripheral neuropathies as an additional method in the substitution of nerve biopsy [20]. Therefore, it is important to understand not only the numerical or morphological variations but also their protein composition of the sensory corpuscles.

All available data on proteins present in human skin sensory corpuscles were obtained using immunohistochemistry techniques, and references for papers reporting each of those proteins are included in the reviews by Vega’s Lab [9,10,19,37,38] and Pawson et al. (Figure 4, Figure 6 and Figure 8) [31].

The axon supplying sensory corpuscles displays immunoreactivity for neuron or neuroendocrine markers such as the protein gene product 9.5 (PGP 9.5) and neuron-specific enolase (NSE). The axon also contains neurofilament proteins.

The glial fibrillary acidic protein (GFAP) could theoretically be considered the intermediate filament protein filling the cytoplasm of the Schwann-related cells forming sensory corpuscles. However, we observed the absence of GFAP immunoreactivity in human cutaneous sensory corpuscles. By contrast, the lamellar cells of the Meissner corpuscles and the inner core of the Pacinian cones display vimentin immunoreactivity. Vimentin was also detected in the outer core and capsule of the human cutaneous Pacinian corpuscles.

Several calcium-binding proteins that participate in mechanoreceptor electrogenesis have been found in human sensory corpuscles. S-100 protein is localized in the lamellar cells of Meissner corpuscles and the inner core lamellae of Pacinian corpuscles. Calbindin D28, calretinin, parvalbumin, and neurocalcin are localized in the central axon and the glial cells of Meissner and Pacinian corpuscles.

Growth factors are substances that stimulate cells to divide or increase in size and have effects on cell differentiation as well as on the acquisition and maintenance of the neuronal phenotype. Several growth factors and their cognate receptors have been detected in human cutaneous sensory corpuscles. The epidermal growth factor (EGF) elicits cell responses by binding to the EGF receptor (EGFR). EGFR immunoreactivity was observed in the axon and lamellar cells of Meissner corpuscles as well as in the axon, inner core, outer core, and capsule of Pacinian corpuscles. On the other hand, neurotrophins (NGF, BDNF, NT-3, and NT-4/5) act on responsive nerve cells binding two types of receptors: p75^LANTR^ (the low-affinity pan-neurotrophin receptor) and tyrosine-kinase receptors encoded by the *trk* family of proto-oncogenes (high-affinity signaling neurotrophin receptors). TrkA, TrkB, and TrkC proteins are the preferred receptors for NGF, BDNFINT-4/5, and NT-3, respectively. In developing and adult human mammalian skin, Meissner and Pacinian corpuscles display p75 and TrkA immunoreactivity, both being highly colocalized. BDNF and TrkB were found in the lamellar cells and the axon, respectively, in the Meissner and Pacinian corpuscles of developing and adult subjects. Interestingly both decreased with age ([25], Vega et al., unpublished]).

Classical neuropeptides were detected in the human cutaneous sensory corpuscles, too. Meissner corpuscles display immunoreactivity for substance P IR, neuropeptide Y, and neurokinin A.

Epithelial membrane antigen (EMA) and glucose-transporter 1 (Glut-1) are present in the cells of the outer core and Pacinian corpuscles. Leucocytary-7 antigen, a myelin-associated glycoprotein, was found in the inner core cells of some Pacinian corpuscles, and myelin basic protein was detected in around 25% of Meissner corpuscles as well as in the preterminal segment of Pacinian corpuscles.

Mechanically gated ion channels are at the basis of mechanotransduction and occur along the somatosensory neurons that reach the skin, including the peripheral extreme tip that forms sensory corpuscles. Moreover, these channels can also be present in periaxonic cells. Thus, deformations in the membrane differ in the cells that form the mechanoreceptors (i.e., axon, glial cells, and endoneurial and/or perineurial fibroblast) and trigger the opening of mechanosensitive ion channels that transduce mechanical energy into electrical activity. The axon of Meissner and Pacinian corpuscles display acid-sensing ion channel 2 (ASIC2), transient receptor potential vanilloid 4 (TRPV4), transient receptor potential canonical 6 (TRPC6), and PIEZO2 immunoreactivity, whereas the inner core of the Pacinian and the lamellar cells of Meissner occasionally show ASIC2 and 4 TRPV4 [37,38].

The extracellular matrix of cutaneous sensory corpuscles is a complex arrangement of molecules filling the spaces between cells, which consist of fibrillary proteins such as collagens and fibronectins and glycosaminoglycans. A major differentiation of the extracellular matrix is the basal lamina, and two main components of it, laminin and type IV collagen, have been detected in human Meisner and Pacinian corpuscles. Interestingly, heparan sulfate proteoglycans were colocalized with type IV collagen in Meissner corpuscles and were located in the outer core lamellae and capsule, but not in the inner core or the intermediate layer, in Pacinian corpuscles [18]. Chondroitin sulfate was observed in the intermediate layer of Pacinian corpuscles but was never colocalized with heparan sulfate proteoglycans and was absent from Meissner corpuscles [16]. On the other hand, the distribution of class I and class II small leucine-rich proteoglycans in human cutaneous Pacinian corpuscles was as follows: the inner core expressed decorin, biglycan, lumican, fibromodulin, and osteoadherin; the intermediate layer displayed immunoreactivity for osteoadherin; the outer core expressed biglycan, decorin, lumican, fibromodulin, and osteoadherin; and the capsule contained biglycan, decorin, fibromodulin, and lumican. Asporin, prolargin, and keratocan were undetectable [17].

## 7. Clinical and Pathological Interest of Cutaneous Sensory Corpuscles and Concluding Remarks

Sensory corpuscles represent the most superficial part of the peripheral nervous system in vertebrates and are therefore easily accessible to be studied. A simple skin biopsy consents the obtention of material to analyze not only the morphology but also the protein composition of the sensory corpuscles, especially the corpuscles of Meissner. In Figure 4 and Figure 6, and illustrating this review, actual knowledge about the proteins identified in the different cell types and the extracellular matrix of human cutaneous sensory corpuscles are included.

Meissner corpuscles survive denervation for even more than 10 years, but denervated laminar cells lack some antigens and change the expression pattern of some others. They also change in morphology and antigen expression after nerve section or entrapment and spinal cord lesion. In recent years, Meissner corpuscles have gained interest since they are absent or reduced in some pathologies involving the peripheral nervous system such as diabetes and HIV-positive patients. In addition, a marked reduction in the number of Meissner corpuscles has been reported in patients with Charcot–Marie–Tooth disease. In multiple sclerosis, axonal degeneration occurs, which reduces the density and structural characteristic density of Meissner corpuscles. Similar findings are found in POEMS syndrome (polyneuropathy, organomegaly, endocrinopathy, monoclonal gammopathy, and skin changes). In some diseases of the central nervous system, abnormalities have also been reported in Meissner corpuscles; for example, a reduction in Meissner corpuscle density in Parkinson’s disease. Reductions in the number of Meissner corpuscles have been reported in spinobulbar muscular atrophy, in Friedreich ataxia, and some psychiatric arteries [19,20].

Pacinian corpuscles in adult subjects survive denervation, showing no structural nor immunohistochemical changes [39]. Tumors involving Pacinian corpuscles are rare and have been defined as Pacinian corpuscle neuroma (or hyperplasia) and neurofibroma [40], which are occasionally painful [41]. Hyperplasia and hypertrophy of Pacinian corpuscles adjacent to digital nerves have been reported, although they are very rare [42,43,44,45].

Therefore, and as a summary, the knowledge of the distribution, morphological characteristics, protein composition, and age-dependent changes in cutaneous sensory corpuscles is of utmost interest to pathologists and dermatologists with a view to using their analysis in the diagnosis and monitoring of treatments.

## Data Availability

Data supporting the images of this Review will be available from the corresponding author upon reasonable request.
